# Airway responsiveness to psychological processes in asthma and health

**DOI:** 10.3389/fphys.2012.00343

**Published:** 2012-09-05

**Authors:** Thomas Ritz

**Affiliations:** Southern Methodist UniversityDallas, TX, USA

**Keywords:** airway hyperresponsiveness, psychological factors, emotion-induction, asthma, stress, psychological

## Abstract

Psychosocial factors have been found to impact airway pathophysiology in respiratory disease with considerable consistency. Influences on airway mechanics have been studied particularly well. The goal of this article is to review the literature on airway responses to psychological stimulation, discuss potential pathways of influence, and present a well-established emotion-induction paradigm to study airway obstruction elicited by unpleasant stimuli. Observational studies have found systematic associations between lung function and daily mood changes. The laboratory-based paradigm of bronchoconstrictive suggestion has been used successfully to elicit airway obstruction in a substantial proportion of asthmatic individuals. Other studies have demonstrated modulation of airway responses to standard airway challenges with exercise, allergens, or pharmacological agents by psychological factors. Standardized emotion-induction techniques have consistently shown airway constriction during unpleasant stimulation, with surgery, blood, and injury stimuli being particularly powerful. Findings with various forms of stress induction have been more mixed. A number of methodological factors may account for variability across studies, such as choice of measurement technique, temporal association between stimulation and measurement, and the specific quality and intensity of the stimulus material, in particular the extent of implied action-orientation. Research has also begun to elucidate physiological processes associated with psychologically induced airway responses, with vagal excitation and ventilatory influences being the most likely candidate pathways, whereas the role of specific central nervous system pathways and inflammatory processes has been less studied. The technique of emotion-induction using films has the potential to become a standardized challenge paradigm for the further exploration of airway hyperresponsiveness mediated by central nervous system processes.

## Effects of psychological processes on the airways—early observations and findings from patients' reports

Observations of airway responses to psychological processes have a long history. Most widely cited are early accounts by Faulkner (1941) who found during examinations of the esophagus, diaphragm, and the bronchial passages that these muscles contracted with unpleasant stimulation. Using bronchoscopy he observed widening and narrowing of the bronchial passages in three patients when he engaged them in conversations about positive and negative emotional topics. Dekker and Groen (1956) reported on cases of patients with asthma who responded with symptoms of dyspnea and lung function decline (verified by serial vital capacity measurements) to provocation with idiosyncratic stimuli, which were associated with memories of highly emotional episodes in their lives. Smith et al. (1970) studied two asthma patients under hypnotic suggestion of anger, fear, and an asthma attack and observed increases in total pulmonary resistance measured with an esophageal balloon catheter under these conditions. These reports are supported by a number of clinical reports and epidemiological studies in which a certain percentage of patients with asthma have endorsed psychological factors as precipitants of their asthma exacerbations (for early reviews, see Weiner, 1977; see also Knapp and Nemetz, 1960; Purcell, 1963; Rees, 1980). However, estimations of the prevalence of psychologically induced asthma exacerbations have varied widely across studies in the past (Weiner, 1977). Most importantly, early observations have also emphasized that asthma patients may experience multiple triggers of symptoms beyond the psychological domain—the notion of a purely “psychogenic asthma” is not well supported by patients' reports and challenge tests that may also generate robust responses to allergen challenges (Dekker and Groen, 1956). Thus, responding of the airways to psychological stimuli has been conceptualized more as a dimensional phenomenon, with some susceptible patients experiencing stress and emotions as predominant factors, others more as subordinate factors (see also Weiner, 1977). These findings are also reflected in studies that have used psychometrically validated questionnaires to capture asthma patients' trigger perceptions. Typically, patients rate multiple triggers with varying degrees of importance and 15–25% of them list various emotions or stress among the top triggers of their asthma (Ritz et al., 2006, 2008). Although largely based on retrospective reports with its inherent biases, these findings provide an important motivation for studying actual airway function under conditions of changing psychological stimulation.

This review will examine observational and experimental research that has studied psychological influences on the airways. Empirical findings will be reviewed on the impact of psychological factors on hyperresponsiveness, the effects of bronchoconstrictive suggestions on the airways, and effects of emotion-induction techniques on airway tone. Potential peripheral and central mechanisms that link psychological processes with airway constriction will then be discussed and recommendations will be given for studying emotion-induced airway constriction using films.

## Observational studies on airway responses to psychological states

Systematic observational studies have confirmed an association between emotional states, mood, or stress and changes in spirometric lung function (for review, see Ritz and Kullowatz, 2005). There seems to be some agreement among longitudinal studies with lung function diaries that negative mood states are associated with reduced lung function. However, studies have also noted considerable inter-individual variability in the association between mood and lung function (e.g., Schmaling et al., 2002). Such individual differences appear to be relatively stable across times of the day (Ritz et al., 2010c). On the other hand, on the group level the overall association of mood states with lung function may be dependent on time of the day and the type of spirometric index used (forced expiratory volume in the 1st second, FEV_1_ vs. peak expiratory flow, PEF). Also, analyzing only extreme episodes of emotional activation may provide more reliable associations between negative affect and lung function decline (Ritz and Steptoe, 2000; Sandberg et al., 2004; von Leupoldt et al., 2006) than analyzing the whole range of possible negative states (Ritz et al., 2010c). Different types of stress or affect can also yield very different associations: in one study, FEV_1_ decreased with changes in current negative affect over a 3-month period, but increased with more daily hassles over the same time period (Kullowatz et al., 2008). Most notably, these changes were mediated by changes in airway inflammation measured by the fraction of nitric oxide in exhaled breath (FeNO).

Strong emotional expressions are sometimes listed in asthma guidelines among precipitants of asthma attacks (e.g., Global Initiative for Asthma, 2010). Laughter and crying are prototypical expressions that have been explored further. Typically, approximately 30–50% of patients report (Gayrard, 1978; Liangas et al., 2003, 2004) airway constriction following laughing. Using PEF diary extracts of situations that involved watching mirth-inducing films in 13 susceptible children, Liangas et al. (2003) observed a 27% fall in PEF after film off-set. Similarly, 40% of children were reported to develop asthma symptoms of wheezing and/or coughing following bouts of crying (Weinstein, 1984), although data from controlled observations are not available. Another naturalistic stressor potentially linked to strong emotional expression, a rollercoaster ride, has been shown to be associated with a reduction in lung function in women with asthma but not in healthy controls, with 39% of the patient sample showing >10% reduction in FEV_1_ (Rietveld and Van Beest, 2006).

## Psychological stimulation under controlled conditions: experimental suggestions alter bronchomotor tone

The most extensive program of controlled laboratory research exploring psychological influences on the airways originated in the 1960s with the development of the bronchoconstrictive suggestion paradigm (for review, see Isenberg et al., 1992). A most typical variant of the experimental setting, the participant is required to breathe through a mouthpiece and tube from a canister, which is introduced as containing a substance that is a powerful bronchoconstrictor. With relative consistency across studies, airway obstruction of a “clinically significant” size (by one or the other criterion, typically more than 15–20% decrease FEV_1_) has been observed in 20–40% of patients with asthma (Isenberg et al., 1992). These airway responses can be blocked by anticholinergic agents, implicating the vagal pathway as a likely mechanism of action (McFadden et al., 1969). Using direct sensitive techniques, such as respiratory resistance measurements by the forced oscillation technique, airway obstruction to bronchoconstrictive suggestions was also observed in participants without lung disease (Kotses et al., 1987a).

In contrast, providing bronchodilatory suggestions alone appears to leave baseline tone largely unaltered (Butler and Steptoe, 1986; Luparello et al., 1970; Put et al., 2004). Nevertheless, a meta-analysis of 33 drug trials found that the average response of the placebo group was a 10% increase in FEV_1_ (Joyce et al., 2000). The smaller effects of dilatory suggestions have been attributed to a floor effect due to the lack of airway pre-constriction: Smith et al. (1970) used hypnotic suggestions to dilate the airways and found that two patients with high initial levels of obstruction indeed demonstrated airway dilations.

Despite its success in demonstrating psychological influences on airway tone, the exact relationship of the suggestion paradigm to emotional processes remains unclear. Although bronchoconstrictive suggestion is sometimes conceptualized as an aversive stimulus (Kotses et al., 1987a), studies have rarely assessed participants' experience of emotion in that context. As an exception, Butler and Steptoe (1986) found that self-reported state anxiety was not affected by this type of suggestion.

## Modification of airway response to physical, pharmacological, and allergic stimuli by psychological factors

A number of studies have demonstrated modification of airway hyperreactivity by psychological factors. Heim et al. (1967) measured carbachol sensitivity by bodyplethysmography in four patients over an extended period and found that independent ratings of “defensive strain” (defined by the authors as a coping situation in which behavior is mounted, largely unsuccessfully, to prevent emergence of threatening or unacceptable behaviors, thoughts, or feelings) were associated with periods of an increase in sensitivity in two of them. A common variant of the suggestion paradigm involves administration of an active stimulus or substance known to affect airway motor tone and studying the effect of suggestion on its potency. Thus, Luparello et al. (1970) observed that presenting a bronchodilator (isoproterenol) as a bronchoconstrictor reduced its bronchodilatory effect (20.1 vs. 39.6% increase in specific airway conductance), and presentation of a bronchoconstrictor (carbachol) as a bronchodilator has been shown to reduce its bronchoconstrictive effects (12.8 vs. 22.3% decrease in specific conductance). In addition, hypnotic suggestions of relaxation, well-being, and exercise without breathing difficulty has been shown to reduce the size of the bronchoconstrictor response to exercise from 31.8% FEV_1_ decrease on control days to 15.9% under hypnosis (Ben-Zvi et al., 1982). Similarly, patients with exercise-induced asthma who had repeatedly experienced symptom relief by pre-exercise inhaler use were also more likely to show less exercise-induced obstruction when subsequently administered a placebo inhaler (visual inspection of the published figure suggested approximately 10% decline in FEV_1_ with placebo compared to 25% without inhaler) (Khan and Olson, 1977). An overview of relevant studies in provided in Table 1.

**Table 1:** **Effects of psychological stimuli on airway responses to pharmacological or physical challenge**.

**Study**	**N (women/men)**	**Challenge type**	**Psychological stimulus**	**Findings**
Heim et al., 1967	4	Carbachol	Observed “defensive strain”	Stronger reactivity in life episodes of strain in two patients
Luparello et al., 1970	20 (13/7)	Carbachol and isoproterenol	Presented as bronchodilator or bronchoconstrictor	Stronger reactivity when presented with correct information
Philipp et al., 1972	20 (6/14)	Acetyl-beta methylcholine	Presented as neutral substance or bronchoconstrictor	Reactivity stronger when presented as bronchoconstrictor
Strupp et al., 1974	13	Isoproterenol	Presented as bronchodilator or bronchoconstrictor	Dilatory effect slightly attenuated when described as constrictor
Ewer and Stewart, 1986	39 (24/15)	Methacholine	Six sessions of hypnotic suggestion of relaxation, ego enhancement, and self-hypnosis	Reduction in reactivity in subgroup of 12 subjects with high hypnotic susceptibility who receive treatment
Pastorello et al., 1987	14 (11/3)	Methacholine	Presented as bronchodilator or bronchoconstrictor	No significant effect
Höglund et al., 2006	16 (10/6)	Methacholine	Academic stress	No significant effect
	19 (10/9)			
Godfrey and Silverman, 1973	7(1/6)	Exercise	Placebo bronchodilator	Reduction in EIA
Heimlich et al., 1966	29	Exercise	Hypnotic suggestion of effortless exercise	Reduction in EIA
Khan and Olson, 1977	32	Exercise	Placebo bronchodilator inhalation	Subgroup of 17 subjects with placebo show reduction in EIA
Ben-Zvi et al., 1982	10(5/5)	Exercise	Hypnotic suggestion of ability to exercise without breathing difficulty	Reduction in EIA
Boner et al., 1988	19 (5/14)	Exercise	Placebo bronchodilator inhalation	Reduction in EIA
Meyer et al., 1990	32 (25/7)	Exercise	Two exercise levels, presented as harmful or not harmful	Bronchoconstriction following higher exercise level when presented as harmful
Liu et al., 2002	20 (11/9)	Allergen	Academic stress	Basic FEV_1_ unaffected, but more increase in sputum eosinphils 24 h later associated with greater FEV_1_ fall
Laube et al., 2003	8 (8/0)	Allergen	Recall of stressful life event	attenuated FEV_1_ fall

Two studies have also explored psychological effects on allergen-induced airway responses. Laube et al. (2003) observed a slight attenuation of FEV_1_ decline to allergen provocation when inhalation of the allergen was followed by recall of stressful life situations (11.2% FEV_1_ decline) as compared to an inhalation procedure without stress recall (15.0% FEV_1_ decline). On the other hand, Liu et al. (2002), using an academic stress paradigm, found a higher sputum eosinophil count and a bias toward Th_2_ cytokine production at 6 and/or 24 h after allergen challenge during the stress period. The fall in FEV_1_ was correlated with eosinophil increase at 24 h after the challenge. Although not addressing airway responses directly, other research in this context has demonstrated the potential of pleasant hypnotic suggestions and mood to attenuate the immediate hypersensitivity response of the skin to histamine (Laidlaw et al., 1996; Zachariae et al., 2001).

A variety of mechanisms can probably account for these different instances of psychological modulation of airway hyperresponsiveness (see also below). To date, little of that has been explored, but it demonstrates that central nervous system activity is capable of changing the outcome of tests that have largely been conceptualized as probing one or the other aspect of peripheral damage or dysfunction at the level of the organ site.

## Airway response to laboratory emotion- and stress-induction

Laboratory studies have also more directly examined airway responses to induced emotions using various standard induction procedures (for reviews, see Isenberg et al., 1992; Ritz and Kullowatz, 2005). Among these were film or picture viewing, imagery, self-referring emotional statements, or recall of autobiographic episodes. With relative consistency, findings suggest a decrease in spirometric lung function or increase in respiratory or airway resistance during negative affective states. Some studies also suggest that eliciting positive emotional states leads to airway constriction (Florin et al., 1985; Ritz et al., 2000; von Leupoldt and Dahme, 2005). Responses to presentation of negative emotional film stimuli specifically appear to yield moderate to high effect sizes (*d* = 0.61 − 1.02 in asthma) (Ritz, 2004). Viewing of film sequences depicting surgical procedures seems to elicit stronger airway constriction than viewing other unpleasant film material (Ritz et al., 2011c, 2012). There is some consistency in individual differences in airway responding across emotion-induction materials and affective qualities (Ritz et al., 2010a). Both asthma patients and healthy controls who respond stronger to surgery films also tend to respond stronger to pictures of blood and injuries. In addition, asthma patients respond stronger to blood and injury pictures also respond more strongly to happy pictures and for healthy subjects this association holds for surgery and amusing films. Table 2 summarizes findings from film stimulation studies in which airway resistance, respiratory resistance, or respiratory impedance were measured throughout films of a negative valence. In one study, we explored changes in airway mechanics during emotional stimulation in greater detail using impulse oscillometry and found that respiratory resistance at 5 and 20 Hz showed airway constriction comparably well, whereas indices of reactance were largely unaffected (Ritz et al., 2010b). This indicates that constriction in the central airways is probably the main source of emotion-induced resistance increases, with little contribution by changes in compliance of the airways.

**Table 2:** **Studies exploring effects of negative emotional film presentation on respiratory resistance**.

**Study**	***N*(women/men)**	**Population**	**Film type**	**Measurement**	**Findings**
Levenson, 1979	29 (18/11)	Adult asthma	Accident film	R_os10Hz_ (during)	Sign. increase for accident and
	12 (7/5)	Adult non-asthma	Asthma-relevant film		Asthma-relavant (sustained)
			Adoption film		Adoption only scene-dependent
Carr et al., 1996	61 (31/30)	Adult asthma	Accident film	R_rs2-32Hz_ (during)	Non-sign. increase for surgery
	18 (12/6)	Adult non-asthma	Surgery film		Accident mixed
	10 (5/5)	Adult asthma panic			
	24 (19/5)	Adult panic			
Ritz et al., 2000	24 (16/8)	Adult asthma	Negative films	R_os10Hz_ (during) sign.	Increase for negative films
	24 (16/8)	Adult non-asthma			
von Leupoldt et al., 2006		Child asthma	Negative films	R_rs5Hz_ (before–after)	No change
Miller et al., 2009	60	Child asthma	Negative film	R_int_ (before–after)	No change
Ritz et al., 2010b	54 (35/19)	Adult asthma	Surgery film	R_rs5&20Hz_ (during) sign.	Increase for surgery films
	25 (16/9)	Adult non-asthma			
Ritz et al., 2011c, 2012	15 (11/4)	Adult asthma	Negative films	R_os10Hz_ (during) sign.	Increase for negative
	14 (10/4)	Adult non-asthma	Surgery film		Asthma-relevant and surgery
	12 (9/3)	Adult blood phobia	Asthma-relevant film		Most pronounced for latter

Note: R_os10Hz_, Oscillatory resistance (equivalent to respiratory impedance) at 10 Hz oscillation frequency; R_rs6-20Hz_, respiratory resistance across multiple frequencies from 2 to 32 Hz; R_rs5Hz_, respiratory resistance from impulse oscillometry at 5 Hz; R_rs5&20Hz_, respiratory resistance from impulse oscillometry at 5 and 20 Hz.

**Figure 1:**
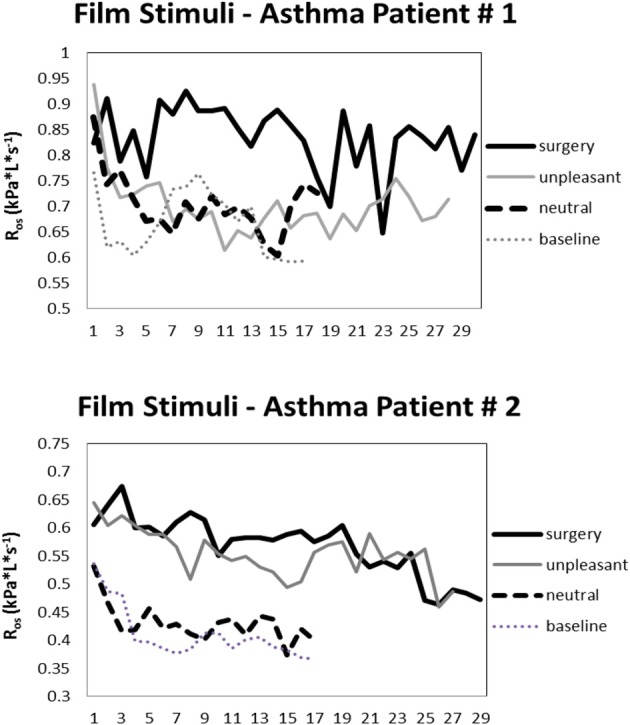
**Oscillatory resistance (consecutive 10-s means) in two exemplary asthmatic individuals during baseline and viewing of neutral, unpleasant, and surgery films**.

Significant differences are sometimes, but not always observed between asthmatic and healthy participants in that the size of the airway response is larger in the former group (e.g., Levenson, 1979; Ritz et al., 2010b). Substantial increases were also found for the announcement of unpleasant films by Levenson (1979). Responses to such films vary in the extent to which they are of a brief, phasic character or are sustained throughout the stimulation period. The latter study observed peaks of airway constriction linked to particular themes or phases of an adoption film and an industrial accident film with an amputation scene. On the other hand, in one recent film study we observed a highly uniform pattern elicited by unpleasant and surgery films in asthma patients and healthy controls, which consisted of an initial increase with a peak within the first 90 s of presentation and then a slow tapering of toward the end of a 4–5 min presentation (Ritz et al., 2012). More sustained resistance increases throughout the film were associated with self-reports of higher arousal and greater shortness of breath after the film. In the first 120 s of surgery film presentation, the average 30 s-interval reached effect sizes of *g* = 1.10 for asthma patients *g* = 0.57 for healthy controls for comparison of surgery with neutral films. Calculating these effect sizes relative to a quiet sitting baseline they reached *g* = 1.47 for asthma patients and *g* = 0.66 for healthy controls. Figure 1 shows oscillatory resistance measured continuously by forced oscillations (single-frequency technique, 10 Hz) throughout baseline, neutral, unpleasant, and surgery films for two exemplary asthma patients. Overall, surgery films elicit the strongest airway constriction across subjects.

Induction of emotions by other techniques has generally confirmed findings with film induction, although effects may be weaker (Ritz, 2004). Increases in resistance are also found with viewing and imagery of negative affective picture material or self-referring depressed statements (Ritz et al., 2001, 2005; von Leupoldt and Dahme, 2005; for review, see Ritz and Kullowatz, 2005). Earlier research using hypnotically facilitated recall of intense states of anger or fear also showed reductions in FEV_1_ of 20% or more in 39% of the tested children with asthma in at least one of six test sessions (Tal and Miklich, 1976). Similarly, Smith et al. (1970) elicited increases in pulmonary resistance in hypnotically induced fear and anger in two adult asthma patients.

Less consistent are findings with laboratory stress tasks, such as interviews, mental arithmetic tasks, free speech, reaction time tasks, or frustrating computer tasks. An earlier study of Mathé and Knapp (1971) measured airway resistance with whole body plethysmography in six individuals with asthma and six controls before and after two tasks: 30 min of frustrating mental arithmetics and a 30-min film depicting accidents and their medical treatment. Resistance was uniformly higher on the stress day than on a control day in asthma, while the reverse was true for controls. In this study, effects of mental arithmetic stress testing could not be disentangled from film viewing effects. Subsequently, Kotses et al. (1987b) demonstrated phasic increases in resistance during brief mental arithmetic tasks in healthy participants using forced oscillation measurements. Further studies found evidence for bronchodilation (Carr et al., 1996) or bronchoconstriction (Ritz et al., 2000) in both healthy and asthmatic participants. Other studies using spirometric assessments showed no substantial changes in lung function following mental arithmetic tasks (Miklich et al., 1973; Aboussafy et al., 2006). Also no significant changes were found in response to a free speech combined with mental arithmetic (Kang and Fox, 2000) and a frustrating computer task (Rietveld et al., 1999) with indices of spirometry. Another study looking at a larger sample (*N* = 114) of asthmatic children only found a trend toward increases in resistance following a 5-min stress interview. Listening to short vignettes of asthma-related scenes was found to decrease PEF by approximately 5% in asthma patients compared to patients with asthma and panic, panic alone, or no diagnosis (Dorhofer and Sigmon, 2002).

*In summary*, emotional stimulation induces mild airway constriction under laboratory conditions. Effects are strongest for unpleasant stimulation material, particularly blood and injury-related films. Positive emotional material has also shown effects in some studies. Constriction appears to be a general response characteristic of the airways to this type of stimulus material regardless of disease status, although some studies have shown stronger effects in asthma patients. Findings with stressful stimuli have remained less consistent.

## Methodological issues in studies of stress- and emotion-effects on the airways

Although an overarching consensus appears to emerge that states of negative affect induce airway constriction or reduce lung function, some inconsistencies remain in particular in the stress-induction literature. Methodological factors such as choice and timing of measurement techniques, type of stress elicited and lack of manipulation success monitoring may at least partly account for some of the inconsistencies (Ritz and Kullowatz, 2005).

### Measurement technique

Forced expiratory maneuvers in spirometric or pneumotachographic assessments do only provide indirect and discrete measures of the airway caliber. They may also enhance bronchoconstriction by irritation of the airways or dilate the airways through deep inhalation, and they require attention and voluntary effort and are thus subject to multiple psychological influences (Smith et al., 1970; Philipp et al., 1972; Spector et al., 1976; Harm et al., 1984). In contrast, respiratory resistance monitoring with forced oscillation or the interrupter technique provides direct and more sensitive assessment of airway caliber and allows for a continuous monitoring of airway status throughout an experimental protocol, which is critical in most studies of emotion (Ritz et al., 2002). Because it relies on tidal breathing it is independent of effort or motivation and does not alter airway tone, and it can thus be expected to produce more valid results. In addition, the multiple-frequency forced oscillation technique also allows a separate estimation of large (central) vs. small (peripheral) airway effects (Smith et al., 2005).

### Timing of assessments

Validity problems may also arise from variations in the timing of measurements which are sometimes inherent in the measurement technique. Discrete spirometric assessments can only be implemented before and after the task as they require attention and effort that would disrupt most tasks. Forced oscillation or interrupter measurements can be implemented for continuous measurements throughout tasks. Measurements need to be restricted to before and after tasks when the character of the task (e.g., involving speech production, use of hands) does not allow for continuous assessments. Two of three film studies that showed no significant change or low effect sizes have relied on pre-post assessments only (Table 2). An additional number of studies that showed weak or no effects have also relied on discrete assessments (Miklich et al., 1973; Rietveld et al., 1999; Kang and Fox, 2000; McQuaid et al., 2000; Put et al., 2004; Aboussafy et al., 2006; von Leupoldt et al., 2006). However, emotions have been conceptualized as phasic, transient events of a short duration (Levenson, 1988). Especially in the artificial laboratory setting an emotional state cannot be expected to continue after task-offset—if anything at all, *recovery* from the emotional state will be measured at this point in time. Indeed, studies that implemented recovery measurements with sensitive techniques have shown that in the first minute following the off-set of stimulation the observed airway constriction is only a weak reflection of that seen during the task (Kotses et al., 1987b; Ritz et al., 2000, 2011c). Taking into consideration that the initiation of a discrete assessment of lung function is time consuming and distracting itself (experimenter instructions, movements to reach for and apply mouth pieces and nose clips, adaptation to the new mode of breathing), such measurements in all likelihood are too far removed from the psychological phenomenon of interest, the resistance change elicited by an emotional state. Discrete measurement protocols operate under the assumption that the clinically relevant state is a tonic constriction of the airways that continues far beyond the actual activation of emotion, an assumption that may not necessarily be valid. The idea may not be ecologically valid with respect to actual dynamics of emotional experience in real life, where periods of emotional turmoil linked to the experience of asthmatic airway constriction may not consist of brief arousal of one emotional state, but of continuous transitions from one negative affective state to the next, or of repeated elicitations of such states in very close temporal proximity.

### Quality and intensity of the psychological state elicited

Given that autonomic outflow is not uniform across emotional states and has been shown to vary along dimensions of affect (Lang et al., 1993) or discrete emotional states (for review, see Kreibig, 2010), it would be simplistic to expect that many different psychological states lead to the same outcome in terms of responding of the airways. Nevertheless, induction of a variety of emotional qualities with standardized induction techniques has been shown to produce constriction of the airways.

For unpleasant affective states, constriction is most uniform across film- and picture induction techniques, which have sometimes been conceptualized as passive coping tasks (Lehrer et al., 1996), because the subject passively endures an aversive stimulus. In contrast, stress tasks often require a more active mode of operation from the subject, requiring the mobilization of information processing capacities, speech, posture changes, or smaller manual activities. Such active action orientation could already mobilize some of the autonomic changes typically seen for these activities, as demonstrated by studies on exercise imagery (e.g., Calabrese et al., 2004). They may also constitute active coping challenges (Obrist, 1976) with strong components of sympathetic arousal that could counteract bronchoconstriction. It also needs to be considered that the emotional quality of such tasks can be mixed or ambivalent and can change with perceived coping success. Classical stress tasks are therefore not comparable with tasks that elicit discrete emotional states more purely, such as carefully pre-evaluated movie scenes (e.g., Gross and Levenson, 1995).

In addition, intensity of the elicited state can also determine airway constriction. Illustrated with a between-individual example, surgery films elicit massive differences in anxiety and disgust experience between individuals with blood-injection injury phobia and asthma patients or healthy controls (Ritz et al., 2011c). Consequently, we observed the strongest respiratory resistance increase in blood-injection-injury phobia individuals (25–30%) compared to healthy controls (11–17%), with asthma patients (21–23%) taking an intermediate position.

It is also notable that hypnotic suggestion tasks often used in earlier studies (e.g., Heimlich et al., 1966; Tal and Miklich, 1976; Ben-Zvi et al., 1982) have elicited considerable bronchoconstriction or bronchodilation (typically 15–20% FEV_1_ change on average) with induction of negative or positive emotional states, respectively, despite mostly using discrete measures of spirometry. It is possible that the emotional states elicited by a hypnotic technique were more intense and in most studies had continued throughout lung function assessments. On the other hand, another study of Clarke (1970) observed FEV_1_ decreases >20% in two of three patients with hypnotic induction of anger and fear only when combined with suggestions of an asthma attack. However, in these studies self-report of the emotional experience was often not measured or not reported in a way that would allow systematic comparison with more recent emotion-induction studies. It is also possible that less sophisticated medication regimens of patients in earlier studies allowed for stronger airway reactivity. A complication of the hypnotic induction is that there are substantial individual differences in susceptibility to suggestion (Leigh et al., 2003), which may make emotion-induction success more variable compared to more recent techniques using films or imagery.

Finally, it needs to be considered that the character of the employed stress challenges has been acute, as compared to more persistent, repeated, or chronic states of stress that are for humans more likely encountered in daily life, rather than in a controlled laboratory setting. Both studies with animals (e.g., Forsythe et al., 2004; Kang and Weaver, 2010) and humans (Kullowatz et al., 2008; Marin et al., 2009) have shown differential responding in airways and asthmatic pathophysiology to states of acute vs. chronic stress. Unfortunately, there is still little consensus in the literature on the exact definition of acute vs. chronic stress challenges.

### Monitoring of manipulation success

From a psychophysiological standpoint, some of the laboratory research on emotion or stress induction and airway responding has been surprisingly limited. Even though, most studies implemented state-of-the-art physiological assessments, the psychological assessment side has been unsophisticated or completely lacking. Thus, only about half of the stress-induction experiments had some type of self-report instruments in place to capture the actual emotional or stress experience of the subjects. Under this scenario, experiential state of the subject is simply being inferred from the potential of the laboratory task to elicit that state, which is reminiscent of an early behaviorist black-box view of higher mental processes (e.g., Obrist, 1976). However, uniform responding of participants to experimental emotion-induction scenarios cannot be expected (Lang, 1994). In addition, given that most modern scientific theories define emotion as a state that unfolds on multiple levels of observation, it appears mandatory to include at least experiential and physiological levels for a minimal description of the phenomenon. Emotion theory would also hold that none of these levels of description can serve as a validation of the other or as ultimate indicator for the presence or absence of emotion (Levenson, 1988). At the minimum, the inclusion of the experiential level would serve as an additional indicator of the validity of the various experimental tasks used in this research and help elucidating their emotional quality. Sole reliance of physiological indices, such as, blood pressure or skin conductance increases, is not sufficient for identifying individuals that respond emotionally to a laboratory stressor (e.g., Horton et al., 1978; McQuaid et al., 2000; Laube et al., 2003).

## Mechanisms of emotion-induced airway constriction

A variety of underlying mechanisms has been proposed for psychologically induced airway constriction. Whereas almost nothing is known about distal central nervous system pathways involved in such responses, at least some studies have explored more proximal autonomic and ventilatory parameters. In exploring such pathways, it will be necessary to keep in mind that even in the realm of emotions and stress, there is sufficient heterogeneity of psychological states and thus in all likelihood heterogeneity in the associated physiological activity (see e.g., Obrist, 1976; Lang et al., 1993; Boiten et al., 1994; Kreibig, 2010). There are also multiple pathways that may result in the same outcome at the end-organ level, i.e., airway constriction, making efforts to find *the* chief mechanism of psychologically induced airway responses a potentially futile exercise. Finally, there are probably a number of different forms of psychologically-induced airway constriction that are linked to differential physiological pathways and that are more or less important in individual patients. The extent of overlap of these phenomena and their clinical implications require further research efforts. The development of standard challenge tests to elicit these responses will be critical for advancing knowledge in this area.

### Autonomic pathways

Perhaps the best evidence to date comes from laboratory studies that have implemented pharmacological blockade. Given that the cholinergic pathway is the main bronchoconstrictor (Canning and Fischer, 2001), efforts have been concentrated on blocking muscarinic impulse transmission at the airway smooth muscle. Already in 1969, McFadden et al. demonstrated that blockade with intravenous atropine abolished airway resistance increases to bronchoconstrictive suggestion. Similarly, Neild and Cameron (1985) blocked bronchoconstrictive suggestion effects in a selected group of patients that had previously shown a clinically significant fall in FEV_1_ to this type of suggestion. This may indicate central vagal excitation or altered sensitivity to normal vagal outflow at the end-organ level. In line with the latter interpretation, Horton et al. (1978) showed a significant association of suggestion effects with hyperreactivity of the airways tested by both methacholine and histamine. Thus, centrally mediated vagal excitation can only be viewed as a major pathway of bronchoconstrictive suggestion effects if airway hyperreactivity to these pharmacological challenges itself is also viewed as at least partially mediated by central vagal pathways (e.g., Haxhiu et al., 2005).

We recently examined airway responses to emotional films and pictures under both placebo and ipratropium inhalation in asthma patients and healthy controls (Ritz et al., 2010b). Increases in resistance were strongest under negative emotional stimulation in patients and were significantly attenuated by cholinergic blockade. Because blockade by ipratropium does not affect cholinergic transmission in the glottis (Widdicombe, 1986), substantial upper airway effects can therefore be excluded as mechanisms of resistance changes. Resistance increases were also not significantly associated with airway reactivity to methacholine, dilatory response to ipratropium at baseline, or basal airway inflammation measured by exhaled nitric oxide. The finding is consistent with that of Lehrer et al. (1996) who did not observe an association between responding to mental arithmetics, reaction time task, or aversive and surgery film viewing with responsiveness to methacholine in asthma patients or healthy controls. Further correlational findings from two studies that have used stimulation with affective pictures and self-referring statement suggest a role of vagal outflow in at least some instances of emotion elicitation. Under conditions of depression induction, respiratory resistance was positively correlated with respiration-corrected respiratory sinus arrhythmia (as index of cardiac vagal activation) in asthma patients (Ritz et al., 2001, 2005). Thus, central vagal excitation appears to be the critical final pathway linking psychological processes to airway responses to viewing of emotional film and picture stimuli.

Modulation of airway tone by sympathetic effects is important, as demonstrated readily by β-adrenergic bronchodilator medication. Action of circulating catecholamines on β_2_-receptors appears to be the primary source of endogenous sympathetic bronchodilation in humans because of a lack of direct sympathetic innervation of the airway smooth muscles (Barnes, 1986; Canning, 2006). However, additional modulation of cholinergic outflow by sympathetic nerves may take place at the level of the ganglia. To date, only sporadic correlational findings are available that may suggest an association of airway responses to emotions or stress with sympathetic activation. Skin conductance, a measure associated with sympathetic cholinergic regulation of sweat gland activity, has shown similar patterns of modulation across emotional states as respiratory resistance (Ritz et al., 2000) and has produced significant positive correlations with respiratory resistance increases between individuals (Butler and Steptoe, 1986; Ritz et al., 2011c). The positive association between an index of sympathetic excitation and respiratory resistance defies simple interpretations and it is unclear whether it signifies an instance of autonomic nervous system fractionation (Berntson et al., 1991), is a sign of a more widespread damage to cholinergic neurotransmission by allergic processes (Kaliner, 1976; Lehrer et al., 1996), or only constitutes a correlation of processes that are functionally unrelated but associated through their relationship with an unknown third variable. Effects of sympathetic excitation may be more prominent in experimental or real-life stressors compared to emotion-induction tasks. Kang and Fox (2000) observed that post-stressor PEF was positively associated with plasma epinephrine levels.

Other autonomic pathways, such as, the nonadrenergic-noncholinergic system have not been studied systematically in humans in the context of stress induction. A role for tachykinins (such as substance P) secreted from sensory nerve endings has been suggested in mice (Joachim et al., 2003, 2004). Sensitized and allergen challenged mice were found to show stronger airway hyperreactivity and eosinophil infiltration following 24 h of sound stress compared to no stress condition, a response that was eliminated with of a neurokinin-1 receptor antagonist. As a hypothetical pathway, stress could enhance activity of the hypothalamic-pituitary- adrenal axis, which has been shown to raise nerve growth factor levels, and that in turn may induce substance P secretion from sensory nerve endings in the airway epithelium. In a cross-sectional study with humans, serum level of brain derived neurotrophic factor has been shown to be elevated in asthma with self-reported chronic stress (Joachim et al., 2008).

A general problem with studying autonomic pathways is a considerable amount of target organ specificity, which has been demonstrated for both parasympathetic and sympathetic systems (Morrison, 2001; Ritz, 2009). Thus, indices of autonomic function derived from other organ sites (e.g., cardiovascular system or sweat glands) cannot necessarily be expected to inform about autonomic regulation of the airways, although target specificity may vary situationally and across states of health and disease.

### Endocrine changes

Given that the airway smooth muscles express receptors for a variety of hormones (Thomson et al., 1996), multiple pathways of influence are conceivable, but research in the context of stress and emotion effects on the airways is still in its infancy. In addition to catecholamines, other endocrine parameters have only been explored sporadically in prior studies. Cortisol levels following an acute laboratory free speech and mental arithmetic stressor were negatively associated with PEF levels in one study with asthmatic and non-asthmatic students (Kang and Fox, 2000), but no association of spirometric lung function changes with cortisol changes from low stress to academic stress periods were observed in atopic or non-atopic students (Höglund et al., 2006).

### Ventilatory changes

*Ventilatory changes* are known to influence airway smooth muscle tone in a number of ways. Deep inspiration is a potent bronchodilator (Nadel and Tierney, 1961; Krishnan et al., 2008), but this effect may be absent in more severe asthma (Scichilone et al., 2007). Airway resistance is inversely related to lung volume and elevations in functional residual capacity are associated with bronchodilation (Forster et al., 1986). Increases in flow can be coupled with increases in airway resistance (Hida et al., 1984), although this association may depend on the behavioral context, with increases in flow related to skeletal muscle activation showing the opposite effect (Baker and Don, 1988). Hypocapnia is also known to constrict the airways through pathways involving autonomic vagal excitation and local airway effects (Newhouse et al., 1964; O'Cain et al., 1979) and this effect is more pronounced in asthma than in healthy controls (van den Elshout et al., 1991). Hyperpnea (or isocapnic hyperventilation) constricts the airways through drying and/or cooling of the airways, which is viewed as the major pathway of exercise-induced bronchoconstriction (McFadden and Gilbert, 1994; Anderson, 2010).

Given that ventilatory influences have often been suspected as a major pathway in emotion-induced airway responding (Clarke and Gibson, 1980; Knapp, 1989), surprisingly few studies have examined such influences. In suggestion studies, only sporadically measurements of timing and volume parameters of the respiratory cycle were included, with two studies showing no changes (Weiss et al., 1970; Butler and Steptoe, 1986) and one showing decreases in tidal volume (V_T_) following bronchoconstrictive suggestion (Put et al., 2004). In emotion-induction studies, ventilatory changes have not been found to be consistently related to changes in respiratory resistance. In one study using unpleasant and surgery-related film material for stimulation, V_T_ changes showed a systematic negative association with resistance, with a 100 ml increase in V_T_ leading to a 0.005 kPa·L^−1^·s decrease in oscillatory resistance, but overall levels of resistance and emotion-induced changes of resistance throughout film clips were not affected by V_T_ change (Ritz et al., 2012).

Ventilatory influences could be most prominent in states involving marked emotional expressions, such as crying (Weinstein, 1984) and laughing (Liangas et al., 2004). Similarly, bronchoconstriction following amusement park rides (Rietveld and Van Beest, 2006) could be related to such factors (screaming, laughing). In addition to airway drying and/or cooling, stimulation of irritant receptors through high flow rates could be another mechanism responsible for such airway constriction in asthma (Hida et al., 1984). However, studies of emotional expression effects have typically not included detailed assessments of the respiratory pattern or gas exchange. Evidence on stress-induced hypocapnia as a bronchoconstrictor has mainly come from early case studies (Herxheimer, 1946). One earlier study observed strong increases in minute ventilation in patients imagining asthma-relevant scenes, but no measures of gas exchange were included (Clarke and Gibson, 1980). In a recent study using a psychosocial laboratory paradigm (free speech and mental arithmetic task under evaluative pressure), no evidence for hypocapnia was found in individuals with asthma or healthy controls during quiet speech preparation and after termination of the task (Ritz et al., 2011b).

### CNS pathways of emotion-induced airway constriction

Animal studies suggest that the airway-related vagal preganglionic neurons in the rostral parts of the nucleus ambiguus and the rostral dorsal motor nucleus constitute the central integrators of multiple CNS influences on airway smooth muscle tone (for review, see Haxhiu et al., 2005). Input is provided from higher CNS centers in forebrain, midbrain and hindbrain, including prefrontal cortex, central nucleus of the amygdala, hypothalamus, and periaqueductal gray (PAG), pons, and nucleus tractus solitarii. A central role seems to be attributed to the PAG, which is also known for its coordination of active and passive coping responses to behavioral challenge. Activation of its ventrolateral portion leads to airway smooth muscle relaxation (Haxhiu et al., 2002) through a GABAergic inhibitory pathway to the vagal preganglionic neurons or through the parabrachial nucleus (Motekaitis et al., 1994, 1996). Another pathway seems to exist from the paraventricular nucleus of the hypothalamus, which has neurons that project directly to the airway-related vagal preganglionic neurons and that augments their activity through release of oxytocin and arginine vasopressin (Kc et al., 2006). Projections from the amygdala to the paraventricular nucleus could then constitute a pathway for inducing bronchoconstriction by negative emotions.

Although there is some exploration of pathways in animal models, little is known about relevant brain regions in humans. Most research stems from studies of respiratory sensation using stimulation by added resistive loads (e.g., Peiffer et al., 2001; von Leupoldt et al., 2009). However, because there is typically only a low to moderate association of respiratory sensations with actual airway constriction and large interindividual differences in this association (e.g., Apter et al., 1997; Ritz et al., 2010c), the value of these studies for informing about CNS pathways of airway constriction is limited. In this context, activation of the PAG has been linked to reduced dyspnea perception (von Leupoldt et al., 2009) and increased gray matter volume of this structure has been associated with longer duration of the asthmatic disease (von Leupoldt et al., 2011). In addition, stimulation of the PAG and the subthalamic nucleus with deep-brain electrodes in patients with movement or pain disorders showed improvements in some aspects of spirometric lung function (14% increase in PEF, but no substantial change in FEV_1_ or Functional Vital Capacity; Hyam et al., 2012). Taken together with findings from animal research, PAG functioning may have a central role in mediating emotion-induced airway responses. Exploring brain regions linked to emotion-induced airway constriction will also require careful consideration of neural pathways involved in ventilatory responses to emotion (Evans, 2010), which will most likely show a certain degree of overlap.

Environmental exposure and disease states of the airways may impact vagal preganglionic neurons or pathways that connect with them and may thus alter CNS processing of various stimuli including those of a psychological character. Prolonged exposure to allergens or noxious agents could damage the GABAergic inhibitory pathway that provides outflow from the PAG (Haxhiu et al., 2002). In a murine model of asthma, evidence was found for a downregulation of noradrenergic inhibitory traffic to the preganglionic vagal nuclei following sensitization and allergen exposure (Wilson et al., 2007). Particulate matter exposure has been shown to reduce excitability of cardiac vagal motor neurons in the nucleus ambiguus of mice (Pham et al., 2009). Exposure to allergens, ozone, or second-hand cigarette smoke in various animal models have been related to plasticity of the nucleus tractus solitarius, which coordinates respiratory motor output of a number of defensive reflexes through vagal pregangionic neurons that may be exaggerated in consequence and thus lead to stronger bronchoconstriction or cough (Chen et al., 2001, 2003; Bonham et al., 2006; Sekizawa et al., 2011). An elevated tendency to constrict the airway in situations that require coping with affective challenges may be the consequence. To date, studies linking CNS pathways with airway responses to emotional stimuli are missing, also because of technical challenges of measuring mechanical lung function in a neuroimaging environment.

Inflammatory processes in the airways related to the late-phase response of allergen challenge, such as increase in sputum eosinophils and reduced glucocorticoid response, have also been shown to be selectively associated with responses of the anterior cingulate cortex and insula to asthma-related compared to neutral words (tested relative to a non-inflammatory methacholine challenge) (Rosenkranz et al., 2005), possibly reflecting the heightened awareness of the inflammatory condition. Bronchoconstriction during the late phase response was also positively associated activation of the insula, which is known for its role in visceral perception.

### Airway immune and inflammatorypathways of emotion-induced airway constriction

Recent human studies have highlighted the role of stress and emotion in modulating systemic markers of inflammation in asthma (e.g., Kang et al., 1997; Marin et al., 2009). The dominant paradigm of this research has been the stimulation of white blood cells to measure cytokine activity in samples taken during stress vs. non-stress conditions as well as asthmatic and healthy subjects, thus essentially informing about the capacity of stress to modulate allergic responses. Following a similar paradigm but studying localized effects in the airways, Liu et al. (2002) demonstrated that late-phase eosinophil levels (measured from induced sputum) to allergen challenge are exaggerated by academic test stress and that such responses are associated with greater bronchoconstriction. On the other hand, purely observational research not involving allergen challenges found that in individuals with asthma, acute negative affect per se was associated with a reduction in FEV_1_, whereas increases in daily hassles across a 3-month period were associated with improvements in FEV_1_ (Kullowatz et al., 2008). These associations were mediated by increases and decreases, respectively, in FeNO as an indirect measure of airway inflammatory status. In this study, findings were only observed with FEV_1_, whereas no association was observed with respiratory resistance measured by impulse oscillometry. This may suggest that experimental emotional stimuli such as brief film clips, which typically show robust but transient increases of respiratory resistance, capture an aspect of emotion-induced airway responding that is different from the constriction observed due to current negative affect, which may also be accompanied by inflammatory processes. The lack of association of film-induced resistance increases with FeNO (Ritz et al., 2010b) is consistent with this interpretation. It is possible that negative emotion-induced airway constriction is based more on phasic change in autonomic outflow, whereas current negative affect as a mood state leads to more tonic change in inflammatory parameters that may also affect the airways and/or only the vigor with which the subject performs the forced expiratory maneuver.

The fast onset of the phasic component of airway constriction to emotional provocation by film stimuli would also preclude a release of bronchoconstrictive mediators from mast cells as underlying mechanism. Corticotropin-releasing hormone secreted from postganglionic sympathetic nerves is known to degranulate peripheral mast cells (Pang et al., 1998; Cao et al., 2005) leading to the secretion of vascular endothelial growth factor (VEGF), which in turn has been linked to bronchoconstriction (Kanazawa et al., 2002). However, airway constriction related to allergen challenge typically develops over minutes rather than seconds (Grainge et al., 2011). These pathways could therefore be more likely involved in tonic airway responses to experimental or real-life stressors. VEGF has been shown to be elevated with negative affect (e.g., Asberg et al., 2009). VEGF from exhaled breath condensate was also found to be increased during academic exam stress in both healthy and allergic rhinitis (including asthmatic) individuals, but only in the latter group were increases in negative affect positively associated with VEGF increases (Trueba et al., 2012). Thus, the role of early-phase response inflammatory mediators secreted from mast cells, including histamine, leukotrienes, and prostaglandins, deserves further scrutiny as a pathway affecting airway tone in acute stress. Additionally, the role of airway nitric oxide, which has been shown to be elevated in response to acute stress and negative affect (Kullowatz et al., 2008; Ritz et al., 2011a), requires further study in this context. Nitric oxide resulting from endothelial and inducible nitric oxidase activity is thought to mediate a range of pulmonary effects of VEGF, including airway hyperreactivity (Bhandari et al., 2006). Nitric oxide has been shown to be associated with either bronchodilation or bronchoconstriction depending on its source (Ricciardolo et al., 2004).

**Figure 2:**
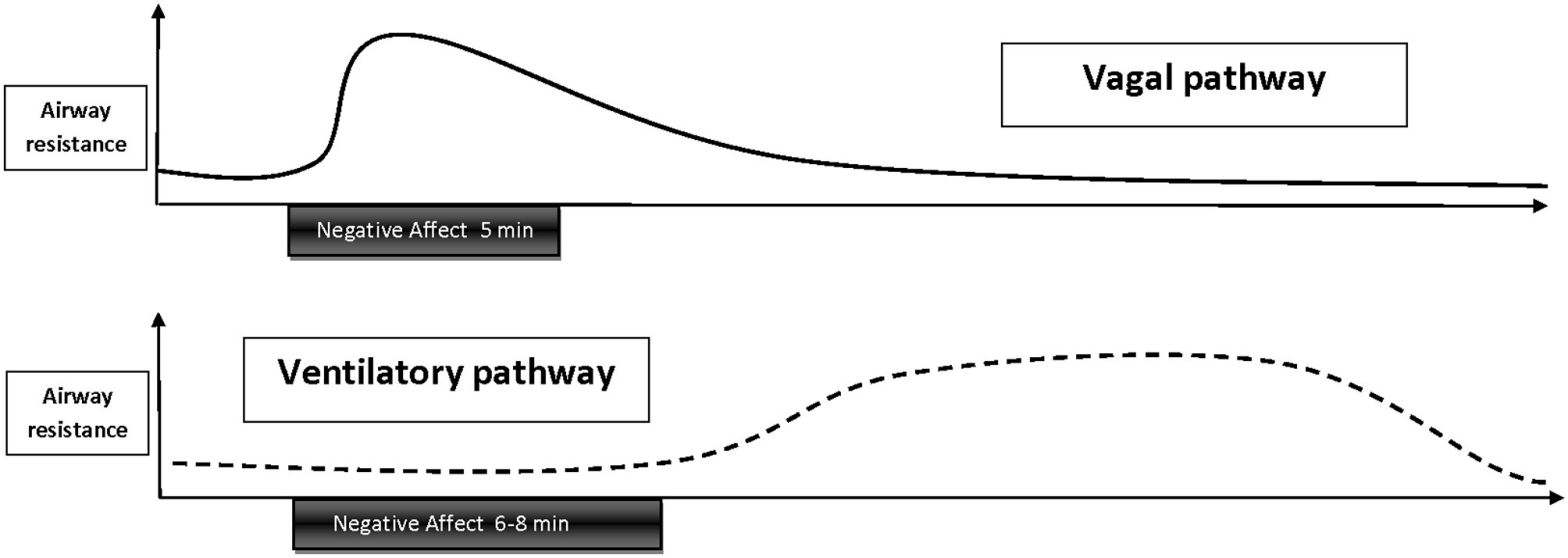
**Schematic depiction of temporal trajectories of emotion-induced airway responses that are thought to be mediated by vagal excitation or ventilatory influences.** Vagally mediated responses have a fast onset, gradually decay throughout emotional stimulation, and subside within 1–2 min following stimulus off-set. Hypothesized airway responses mediated by ventilatory changes, such as hyperpnea, would follow a similar trajectory as bronchoconstriction induced by exercise—or cold air hyperventilation, with a delayed onset during the late phase of a longer (6–8 min) emotional stimulation or after off-set of stimulation and persistence of constriction over 20–40 min.

*In summary*, at the present stage of research the two most plausible pathways linking emotion or stress with bronchoconstriction are autonomic vagal and ventilatory influences, in particular airway cooling and/or drying. It should be noted that these pathways would likely result in different temporal characteristics of bronchoconstriction (Figure 2). Airway constriction by vagal excitation would show a fast onset within the first 10–20 s of the beginning of stimulus presentation and subside with 1–2 min of stimulus off-set. Constriction due to emotion-induced hyperpnea would be more likely to follow a pattern similar to exercise or cold air challenges, which are typically administered for 6–8 min (Weiler et al., 2010), but the intensity of the constriction would depend on the level of ventilation the person reaches. So far empirical evidence has only been provided for the first type of response trajectory. Little is known about the dynamics and mechanisms of tonic components of airway constriction to emotion or stress and other temporal characteristics such as cumulative effects of repeated stimulation, sensitization, or habituation.

## Clinical relevance of emotion-induced airway obstruction

Indicators of the importance of psychologically induced airway constriction could be the overall intensity of constriction elicited, its association with asthma-related symptoms experienced in that moment, or its association with patients' experience of emotion-induced asthma symptoms or airway obstruction in daily life. With respect to intensity, large effects sizes typically observed for resistance changes (Ritz, 2004; Ritz et al., 2010b, 2011c) within subjects are one possible metric (see also above). Cut-off scores for percent change in lung function are more often used in the clinical context, but are only well-established for spirometric indices that are problematic in the context of emotion-induction research (as outlined above). They are also somewhat arbitrary, with e.g., suggestions for anything between 6 and 25% decrease in FEV_1_. (e.g., Weiler et al., 2010) for exercise-induced bronchoconstriction. In one ambulatory study that utilized FEV_1_ to capture lung function decline in extreme daily life negative mood states we found that 25% of individuals with asthma showed FEV_1_ decreases ≥15% (Ritz and Steptoe, 2000). To evaluate changes in resistance, we have used evidence from added resistive load studies on typical thresholds for just noticeable differences, which are in the area of 0.076 kPa^*^L^*^s^−1^ for asthma patients (Dahme et al., 1996). Across studies, we found that 12.5–29% of patients exceeded this criterion with their resistance increases to unpleasant film stimuli (Ritz, 2004; Ritz et al., 2010b, 2011c). Associations between resistance increases and symptoms have typically been low and insignificant when resistance averages over the whole film duration were computed. However, an analysis of trajectories of resistance change across unpleasant and surgery films has shown that more sustained constriction, rather than initial peak increases with subsequent fall of respiratory resistance, were predictive of subsequent shortness of breath reports (Ritz et al., 2012). Of note, peak airway constriction to this type of film stimulation reached >30% increase in respiratory resistance (roughly equivalent to 12% FEV_1_ fall, see Footnote 5 in Ritz et al., 2012) in at least a quarter of the tested asthma patients.

Another indicator of the clinical relevance of negative affect induced resistance changes is the association between laboratory and field assessments of lung function decline. Two studies have explored this association so far. Ritz and Steptoe (2000) found that respiratory resistance (by forced oscillation technique) increases to negative films, in particular one that induced sadness/depression, in the laboratory were significantly associated with FEV_1_ decline in episodes of strong negative mood during a 3-week lung function diary assessment in 20 individuals with asthma, but not 20 healthy controls. Another study using a smaller sample of asthma patients and controls (*n* = 10 each; von Leupoldt et al., 2006) also found negative but non-significant associations between airway resistance measured by body plethysmography increases during unpleasant picture material and PEF decline during extreme negative mood episodes of a 3-week lung function diary assessment. Studies with larger sample sizes and longer field assessment periods to capture more episodes of extreme mood states would be indicated to consolidate findings in this area.

**Figure 3:**
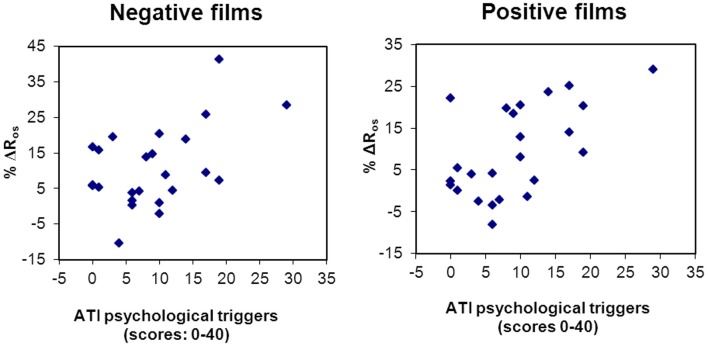
**Scatter plots of the association between self-report of asthma emotional triggers in daily life and respiratory resistance change to laboratory emotion-induction using films of a negative (*r* = 0.48, *p* = 0.017) and positive (*r* = 0.58, *p* = 0.003) emotional valence (Ritz et al., 2006)**.

Finally, studies have explored the association between psychologically induced lung function decline in the laboratory with patients' reports of psychological asthma triggers. Specific airway resistance increases to bronchoconstrictive suggestion appear to show little substantial association with the experience of emotion-induced asthma symptoms (Janson-Bjerklie et al., 1986). On the other hand, Tal and Miklich (1976) found that decreases in FEV_1_ following an autobiographic recall of anger and fear episodes in asthmatic children were associated with prior reports of emotion-induced asthma in daily life. Similarly, patients' reports of a greater importance of stress and emotion as daily life asthma triggers were associated with respiratory resistance increases to unpleasant and pleasant films in one study (Ritz et al., 2006; Figure 3), resistance increases to surgery films in a second study (Ritz et al., 2010b), and to more sustained increases resistance throughout surgery films in a third study (Ritz et al., 2012). Thus, experimental emotion-induction by films appears to be an ecologically valid paradigm for studying emotion- and stress-related airway obstruction.

## The film paradigm for eliciting emotion-induced airway obstruction—recommendations

Films have been widely used for experimental induction of emotions (Rottenberg et al., 2007). Given the focus of commercial movies on dramatic entertainment, they are an excellent source of emotion eliciting material. Among the advantages of this technique is that film sequences can be presented in a standardized form and used across laboratories for replication. Pre-evaluation of the emotional content of the material in independent samples using standard rating scales is necessary to determine the validity with respect to emotion elicitation.

A number of studies have provided suggestions and instructions for extracting materials and have generally shown good success in eliciting target emotional states (e.g., Philippot, 1993; Gross and Levenson, 1995; Rottenberg et al., 2007). A number of methodological consideration need to be made when implementing the technique in a laboratory study.

In summary (see also above):

### Continuous measurement

In order to study airway dynamics during changing experiential and behavioral conditions, constriction should be monitored throughout film presentations while the emotional process unfolds. Forced oscillation and interrupter techniques are well suited for this purpose (Ritz et al., 2002). Measurements following off-set of the stimulation material may be insensitive and conceptually problematic because they capture effects of recovery from stimulation rather than the effect of stimulation itself. Lung function assessments that require a forced expiratory maneuver are not suitable because they are discrete rather than continuous and interfere with the phenomenon of emotion.

### Monitoring of respiration

Additional monitoring of ventilation is strongly recommended to explore potential ventilatory mediation of resistance increases, by e.g., reductions in V_T_ or hypocapnia.

### Duration of the presentation

Peak response is reached within the first 1–2 min of presentation, thus, film sequences as brief as that may suffice; however, trajectories of constriction across longer presentations (up to 5 min) have also been shown to provide useful information (Ritz et al., 2012).

### Choice of baseline

As a reference, neutral film material is preferable, as it controls for non-specific effects of film viewing as such and helps to isolate experimentally the emotional aspect of the stimulation material. To control for within-session changes in tonic levels of airway resistance, it may be advantageous to implement intermediate baselines or brief pre-presentation measurements of 1–2 min duration.

### Evidence for manipulation success

Self-report of emotion should always be captured in addition to resistance monitoring, because it provides concurrent evidence for manipulation success. Video monitoring of facial expression (Ekman and Friesen, 1978) or electromyographic recordings of activity over facial muscle sites (Fridlund and Cacioppo, 1986) is an excellent additional level of behavioral evidence, although technically more demanding and sensitive to audience effects (imagined or real presence of individuals co-viewing the material with the participant; Fridlund, 1991; Hess et al., 1995).

### Capturing participants' experience

Emotion is a construct with multiple facets that are not necessarily highly correlated. Rating of emotional states should therefore employ measures that reflect this plurality of states. The two dominant models of the structure of emotion either distinguish discrete emotional states (e.g., anger, sadness, and joy; Izard, 1991) or dimensions of emotion (e.g., valence and arousal; Russell, 1980; Lang et al., 1990). Using both approaches to measurement in the same study may be advantageous because co-variation of respiration and respiratory resistance has been observed with both models of emotion (Boiten, 1998; Ritz, 2004; Gomez et al., 2005). Respiratory sensations are another important domain of experience to be measured in studies of airway psychophysiology (Ritz et al., 2002). A solid body of literature has demonstrated multiple facets of respiratory sensations or dyspnea that require multidimensional assessment strategies (e.g., Kinsman et al., 1973; Simon et al., 1989; Lansing et al., 2009; Parshall et al., 2012).

### Content of films

Although there is evidence that a wide range of emotional states leads to airway constriction, film material of the blood-injection-injury type, or scenes of bloody surgery from medical education material, appears to be particularly potent (Ritz et al., 2011c, 2012). This also applies to individuals with asthma, who do not respond stronger to film scenes of asthma symptoms or attacks than to various other types of unpleasant film scenes.

### Withdrawal periods for medication

Bronchodilatory medication should be discontinued prior to the assessment. At least 8 h have been recommended for short-term bronchodilators, 16 h for long-term bronchodilators, and 24 h for leukotriene modifiers (Ritz et al., 2002). Longer wash-out periods of up to 1 week will be needed for Tiotropium, a long-term anticholinergic agent that shows extremely slow dissociation from M_3_ muscarinic receptors (Barnes et al., 1995).

### Control for psychological characteristics as moderators of airway responding

Stable psychological characteristics, such as, previous experience of psychological asthma triggers (Ritz et al., 2006, 2010b, 2012), self-efficacy in asthma management (Campbell et al., 2006), or a tendency to use defensive coping strategies (Feldman et al., 2002) have been shown to moderate effect of emotional stimulation on the airways. Controlling for these and other psychological trait and temperament variables is recommended for future studies. Similarly, moderator effects of comorbid psychiatric disorders, particularly anxiety and mood disorders, on emotion-induced airway responses require further study, as two studies of comorbid asthma and panic disorder have demonstrated (Carr et al., 1996; Dorhofer and Sigmon, 2002).

## Conclusion and outlook

Psychologically elicited airway responses have been well described in the literature and can be elicited with standardized laboratory challenges. The airway response to such tests provides clinically relevant information about emotion-induced asthma, a condition with a high prevalence and associated costs for asthma control and quality of life (Ritz et al., 2006, 2008). Psychological challenge protocols could be a meaningful extension of the available battery of direct and indirect airway challenges available to clinicians. There is a growing realization that psychosocial factors influence the pathophysiology of the airways in asthma (Bienenstock, 2002; Marshall, 2004; Vig et al., 2006; Wright, 2010; Douwes et al., 2011). Vagally mediated airway constriction that accompanies affective processes may play a central role in this context.

Future research must further elucidate the relationship of emotion-induced airway responses with those to various indirect and direct challenge procedures (Crapo et al., 2000; Joos et al., 2003). Phasic and tonic components of responding require further exploration, as does the relationship with acute and late phase responses of the allergic cascade and different asthma endotypes and phenotypes that have been proposed recently (Wenzel, 2006; Lötvall et al., 2011). The relationship to structural changes in the airways by disease-related remodeling (Busse, 2010; Al-Muhsen et al., 2011) will also need to be explored. In addition, expanding our rudimentary knowledge on CNS pathways will be necessary in coming years. Beyond asthma, the role of psychologically induced airway responses in other respiratory conditions, such as, viral airway infection or COPD, requires further exploration. Finally, treatment approaches that target these forms of airway responding are still missing. Anticholinergic therapy may be a viable pharmacological approach, but behavioral intervention could provide more sustained and preventative long-term effects. Interdisciplinary research efforts in a behavioral medicine context are needed to advance this research agenda.

### Conflict of interest statement

The author declares that the research was conducted in the absence of any commercial or financial relationships that could be construed as a potential conflict of interest.
